# Elixers and Inoculations

**Published:** 1890-02-22

**Authors:** 


### The Doctor's Pulpit.

### ELIXIRS AND INOCULATIONS.

In the *Argus* of November 4th, 1889, a story appeared which purported to be the confession of a miserable wretch who had sought to renew his youth by means of inoculation with the new elixir of which the public heard so much a short time ago. The story has a certain importance, as showing the peculiar sensibilities excited in the non-professional mind by the idea of restoring youth by such means. It is bare justice to the medical profession in this and other countries to say that Dr. Brown-Sequard's supposed discovery not only aroused in its members no enthusiasm, but, on the contrary, excited chiefly disgust and contempt. It is quite possible that there may be among doctors men of crude and unphilosophic mind, who imagine that nature has secrets which, if they could but be discovered, would not only abolish death, but supply to those who could pay for them the fires and energies of perpetual youth. This is only the same as saying that, among doctors as among other people, there are those in whom imagination is stronger than reason, and passion more powerful than conscience.

The practice of inoculation, whether for prophylaxy, cure, or the restoration of vital energy, is one which urgently demands the consideration of competent scientific men at the present time. It is not a new practice, but it is new in the extent to which it has been carried out during the last few decades. Vaccination against smallpox of course furnishes the most familiar example of inoculation for a definite health purpose; and this practice has received the almost universal assent of medical men in every civilized country during the last half century. More recent than vaccination for smallpox is M. Pasteur's practice of inoculation for the cure of rabies. Newest of all is Brown-Sequard's discovery, or re-discovery, of the secret of immortal youth.

It is of the profoundest possible concern, both to medical men and to all other human beings, to search with the utmost thoroughness for all kinds of knowledge which bear upon so striking a departure from physiological experience and precedent as is implied in the practice of inoculation. The average medical man, and indeed the average man of every other class, seems to think that because inoculation has been extensively practised for a century, its reasonableness and scientific value are thereby established beyond doubt or question. If that be indeed so, then surely there is before the medical profession a field of discovery almost infinite in extent. If we inoculate for smallpox, why not for every other disease of an exanthematous character? And if the poison of rabies can be neutralized and destroyed by inoculation, why not every other poisonous germ, whether of typhoid, typhus, diphtheria, or any other microbe whatsoever? If inoculation as a general principle of prophylaxy and cure can do what is claimed for it, then surely we are but at the beginning of a journey, whose end is in the happy region of perfect health, and of all but immortal youth!

But we affirm in the boldest and clearest of tones that inoculation, whether for the prevention of smallpox or for the cure of rabies, has not yet demonstrated its value to every instructed and reasonable mind. This is not the same as denying the prophylactic efficacy of vaccination, or the curative energy of inoculation for rabies. But it is affirming that the medical men who established the practice of vaccination a century ago had neither the scientific capacity nor the historical aids for settling a question of this kind with such a degree of conclusiveness that it should never again need to be reopened for all time. The scientific capacity of the medical men of to-day far exceeds that of their predecessors of the last century, whilst the accumulated medical history of the period between Jenner and the present affords bases of research and means of comparison which were entirely unknown to Jenner and his contemporaries.

We are not now making any protest against vaccination, or even against Pasteurism. But we are contending, and with all possible energy, that science and philosophy alike demand the reconsideration of the whole subject of inoculation by the competent medical scientists of the present time. Is it such a strange thing in the history of either philosophy or science that errors should be established and should retain a permanent hold upon the human mind for a period of a hundred years? If all errors hoary with the age of a thousand years could be at once and for ever destroyed how much happier and more prosperous the world would be! What schoolboy has not heard of the Ptolemaic and Copernican systems? Who is not familiar with the Mosaic Cosmogony? But to come nearer home, what doctor does not blush, even to the very floor of the fourth venticle, when he remembers the stupidities, the imbecilities, the barbarities, and the disgusting brutalities practised by his predecessors even since the days of Bacon? What is the history of philosophy from the times of Plato and Aristotle until now? Did the Stagyrite take pleasure in agreeing with his master; and did his own immediate disciples always understand what he taught, or practice even so much as they understood? Were the Stoics wisest in their views of life; or did the Epicureans point out a better way? How wondrous were the contradictions of the schoolmen, and the still more striking diversities of opinion among the fathers of the Church! If Aquinas was right, what a heretic was Abelard! If Augustine's orthodoxy was scientifically sound, who will answer for Pelagius? To come down to recent days, we have Hume and Hamilton, Paley and Whewell, Bentham and Reid, Mill and Calderwood. Confusions, contradictions, misapprehensions, and interminable contentions mark every page in the history of philosophic speculation. And does medicine, the latest of the sciences, that was born but yesterday, expect to avoid the snares and pitfalls that lie in the way of human progress always and everywhere? If a study of speculative philosophy disposes the mind to ask whether it be possible that such a thing as truth can be found in the world, what must be the state of mind of him who reads through the history of only the last ten centuries of European medicine?

The truth unhappily is that original minds are exceedingly rare, and probably rarer among doctors than among other classes. The average mind both of the scholar and of the fool hates originality. It has never found itself the worse off for want of that peculiar commodity; and it cannot understand why any other mind should fail to be content with things as they are. But the present period of medical history demands nothing so much as originality of mind. It is not mere experimentation on animals that is wanted now; that, indeed, has ceased to require originality. What is needed is the intellectual power of seeing familiar things as if they had never been seen before; of seeing them in their true magnitude and relation; of seeing them in the full light of all past history and speculation; of seeing them in the noonday blaze of all the science of the present time. Doctors are swamped by prejudices, blinded by custom, hide-bound by "use and wont." If they could but see this whole question of inoculation in its true light, and as it strikes the vast world that knows nothing of medical ways, their instinctive loyalty to true science would compel them to immediate action. They would give themselves and each other no rest until the most competent medical scientists of the time should have at least used their best efforts to demonstrate whether or not the human race ever has obtained or ever can obtain permanent advantage from any kind of inoculation whatsoever.

